# Temperature Increase and Damage Extent at Retinal Pigment Epithelium Compared between Continuous Wave and Micropulse Laser Application

**DOI:** 10.3390/life12091313

**Published:** 2022-08-26

**Authors:** Yoko Miura, Keiji Inagaki, Alessa Hutfilz, Eric Seifert, Benedikt Schmarbeck, Akira Murakami, Kishiko Ohkoshi, Ralf Brinkmann

**Affiliations:** 1 Institute of Biomedical Optics, University of Lübeck, 23562 Lübeck, Germany; 2 Medical Laser Center Lübeck, 23562 Lübeck, Germany; 3 Department of Ophthalmology, University of Lübeck, 23562 Lübeck, Germany; 4 Inagaki Eye Clinic, Chiba 279-0011, Japan; 5 Department of Ophthalmology, Faculty of Medicine, Juntendo University, Tokyo 113-8421, Japan; 6 Department of Ophthalmology, Hiroo Hanezawa Internal Medicine and Ophthalmology Clinic, Tokyo 150-0012, Japan; 7 Department of Ophthalmology, St. Luke’s International Hospital, Tokyo 104-8560, Japan

**Keywords:** retinal pigment epithelium, minimally invasive retinal laser treatment, continuous wave laser, micropulse laser, duty cycle, temperature increase

## Abstract

Continuous wave (CW) and microsecond pulse (MP) laser irradiations were compared regarding cell damage and laser-induced temperature rise at retinal pigment epithelium (RPE). The RPE of porcine RPE-choroid-sclera explants was irradiated with a 577 nm laser in CW or MP mode (5% or 15% duty cycle (DC)) for 20 ms or 200 ms at an average laser power of 20–90 mW. Cell viability was investigated with calcein-AM staining. Optoacoustic (OA) technique was employed for temperature measurement during irradiation. For 200 ms irradiation, the dead cell area (DCA) increased linearly (≈1600 µm^2^/mW) up to the average power of 40 mW for all modes without significant difference. From 50 mW, the increase of DCA of MP-5% significantly dropped to 610 µm^2^/mW (*p* < 0.05), likely due to the detected microbubble formation. OA temperature measurement showed a monotonic temperature increase in CW mode and a stepwise increase in MP mode, but no significant difference in the average temperature increase at the same average power, consistent with the temperature modeling. In conclusion, there is no difference in the average temperature rise between CW and MP modes at the same average power regardless of DC. At lower DC, however, more caution is required regarding mechanical damage due to microbubble formation.

## 1. Introduction

More than 10 years have passed since so-called subthreshold retinal photocoagulation therapies claiming minimal or even no damage to the tissues were introduced to treat macula diseases [1,2]. Mainly two approaches are used clinically: the non-damaging endpoint therapy reduces the irradiation time of the continuous wave (CW) mode of typically 100–300 ms by one order of magnitude and suppresses heat flow further away from the application area [3]. The micropulse system applies repetitive microsecond pulses (MP) with breaks in between and claims to provide a milder temperature increase at and around the retinal pigment epithelium (RPE) than CW irradiation [4]. In fact, the MP mode has been shown to be effective in the treatment of different macular diseases such as central serous chorioretinopathy (CSC) [5,6,7,8] and diabetic macular edema (DME) [9,10,11,12]. However, a universal understanding of the correct mechanisms of action has not been achieved yet and controversial discussions continue. A proper understanding of the difference and similarity between CW and MP modes is essential for the effective use of both modalities for its intended use for certain diseases.

One of the main issues refers to the temperature increase and associated cell damage and damage range between CW and MP mode. Therefore, the aim of this work is to gain more insight here by measuring both for the two different application modalities. The principle of the MP is the irradiation in the form of pulses with repetitive “on” and “off” times. The durations of these “on” and “off” times are variable by defining duty cycle (DC), typically used from 5 to 15% by keeping the repetition rate constant at typically 500 Hz [13]. Thus, the single pulse durations (duration of each on-time) vary from 100–300 µs in the DC range mentioned. In the clinical practice, an overall irradiation time of 100–200 ms is widely used. The average laser power is thus the pulse power multiplied by the DC, and the total energy delivered to the irradiated tissue is the average power multiplied with the overall irradiation time. In case of the conventional CW laser irradiation, the pulse power and average power are identical since the DC is 100%.

The damage of tissue from heat exposure is determined by the temperature and the time the elevated temperature is present. It can be approximated by the Arrhenius theory of thermal damage [14]. While the temperature increases monotonously in a CW irradiation, a stepwise temperature rise (during the “on” time) and fall (during the “off” time) occurs in the MP mode. However, the average temperature rise is supposed to be almost identical to the CW mode, as it has been calculated by Wang et al. [15]. They also showed that the thresholds for tissue effects observed by fundus and fluorescein angiography on rabbit eyes were largely defined by the average power regardless of DC. The results were supported by heat diffusion and damage range calculations by means of the Arrhenius model [15].

At present, there are still no studies comparing the area of RPE damage for the different application modes. Further experimental temperature measurements during irradiation are missing in order to validate the theoretical results. Therefore, and as the aim of this study, we performed experiments determining the extend of RPE damages by CW and MP laser irradiations with the same average power and same total irradiation time using porcine RPE explants. Further we measured the temperature rise during irradiation by optoacoustic method as has been demonstrated for photocoagulation earlier [16]. The temperature courses were also compared to the theory based on an appropriate absorption model and heat diffusion calculations.

## 2. Methods

### 2.1. Laser System and Power Measurement

An IQ577^®^ laser system (IRIDEX corporation, λ = 577 nm, Power range: 50–2000 mW) was utilized for the study. This laser system enables laser irradiation in the CW mode as well as in the MP mode operating with micropulse repetition rate of 100–1000 Hz (the most clinically used is 500 Hz), a variable DC, and an adjustable irradiation time per spot.

A photodiode (PDA10A-EC, Thorlabs, Inc., Newton, NJ, USA) was used to determine the temporal laser profile with a digital oscilloscope (Tektronix GmbH, Köln, Germany) and to determine the exact repetition rate of laser radiation. Prior to the experiments, a calibrated power meter (Ophir Optronics GmbH, Nienburg/Weser, Germany) was used to measure the average laser power at the output of the laser link, that is, the averaged laser power reaching the tissue. Based on this measurement, correction factors for each mode were determined so that the set power can be determined to obtain the desired power at the tissue. All subsequent descriptions of laser power in this document refer to the laser power applied to the tissue.

### 2.2. RPE Organ Culture

The study was conducted using RPE-choroid-sclera explants from freshly enucleated porcine eyes obtained from a local slaughterhouse. After the extraocular tissues were removed, the eyes were immersed in a disinfection solution (povidone iodine, diluted to 2%) for about 5 minutes, rinsed with sterilized phosphate-buffered saline without calcium and magnesium (PBS(−). The anterior part of the eye was resected through the circular incision at approximately 5 mm behind the corneal limbus, followed by the removal of the lens and the vitreous. A round piece of tissue composed of the retina-RPE-choroid-sclera were extracted using a trepan with a 12 mm diameter and a scalpel. This tissue explant was immersed in the sterilized PBS(−) and the neural retina was carefully removed from the RPE layer. The remained RPE-choroid-sclera explants were maintained in Dulbecco’s Modified Eagles Medium (DMEM; high glucose, Sigma Aldrich, St. Louis, MO, USA) supplemented by 10% porcine serum, 1 mM sodium pyruvate, 100 unit/mL penicillin and 0.1 mg/mL streptomycin (Sigma Aldrich) at 37 °C in 5% CO_2_ incubator. After incubating the tissues for 24 h, the laser irradiation was conducted.

### 2.3. RPE Cell Viability Test

For visualization and discrimination of live and dead RPE cells after laser treatment, RPE cell viability was investigated with calcein-acetoxymethylester (AM) (Thermo Fisher Scientific, Waltham, MA, USA). Calcein-AM is a non-fluorescent permeable dye, which can be converted to the green-fluorescent calcein in the presence of intracellular esterase through the acetoxymethyl ester hydrolysis. One hour after laser irradiation, the explants were incubated with 10 µM calcein-AM (in PBS(−)) for 30 min. After being washed with PBS(−), the RPE was observed with a fluorescence microscope (Eclipse Ti, Nikon, Tokyo, Japan) using a filter for λ_ex_/λ_em_ = 465–495 nm/515–555 nm. In the living cells, the dye may be well retained in cytoplasm, and thus, they are fluorescent. On the other hand, the dead cells with high cell membrane permeability were detected as non-fluorescent. For each laser spot, the area of non-fluorescent cells (dead cell area (DCA) (µm^2^)) was measured with an open-source software Fiji.

The DCA among three modes was compared by one-way Analysis of Variance (ANOVA) with Tukey’s multiple comparison, using the statistic software PRISM (Ver. 7.04, GraphPad Software, Inc., San Diego, CA, USA). A *p*-value less than 0.05 was determined as significant.

### 2.4. Laser Irradiation

The experiments with respect to the RPE cell damage in the CW and MP-modes can be divided in two parts. Part I was used to measure damage areas at the RPE with different laser settings. Part II was designed to perform temperature measurements using an optoacoustic technique during CW and MP-irradiation on the explants with a modified set-up. In order to support and analyze the findings, temperature calculations were performed using an appropriate thermal diffusion model.

#### 2.4.1. Part I: Setup for Determination of Damage Areas and Thresholds

**Figure 1:**
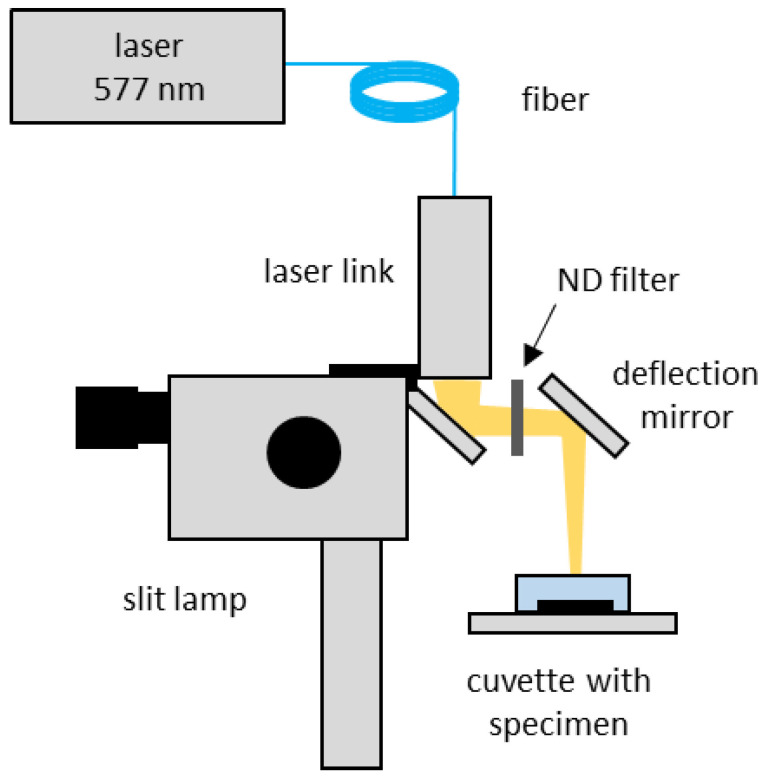
Experimental Set-up for RPE damage detection.

In order to perform the irradiation on the samples in the culture dish located horizontally as shown in Figure 1, a deflecting mirror (CCM1-P01/M, Thorlabs, Newton, NJ, USA) was fixed at an angle of 45° in front of the slit lamp with the laser link.

Laser irradiation on the RPE was performed 24 h after tissue preparation. For irradiation, the tissue was placed in a culture dish (33 mm diameter) with PBS(−) with the RPE side up. PBS(−) is optically transparent and does not absorb laser light.

In this study, the RPE was irradiated with a total of nine average powers (20–90 mW: 20, 30, 35, 40, 50, 60, 70, 80, 90 mW) in 3 different modes, CW, MP with 15% DC (MP-15%), and MP with 5% DC (MP-5%), with irradiation time of either 20 ms or 200 ms. These parameters correspond to an irradiation energy per spot from 0.4 to 1.8 mJ and 4 to 18 mJ for t = 20 ms and 200 ms, respectively. Using the correction factors described earlier, the set power to obtain the desired average output power was determined and verified to be correct. In order to enable the irradiation with a power lower than 50 mW in the CW mode, a neutral density (ND) filter with an optic density of 0.6 (25% transmittance; Edmund Optics GmbH, Mainz, Germany) was placed on the beam path in front of the deflection mirror.

The laser was applied on the explant RPE so that spots irradiated in the same mode were aligned vertically and those irradiated at the same average power were aligned horizontally. The base temperature was the room temperature, around 22 °C. For each irradiation duration, at least six explants were examined.

#### 2.4.2. Part II: Temperature Determination during Irradiation

##### Optoacoustic Temperature Measurement

In order to assess the temperature rise during irradiation an optoacoustic method was used, which is described in detail elsewhere [16,17]. In short, the method makes use of the temperature dependence of the Grüneisen coefficient, which describes the thermoelastic expansion of tissue during heating. A pressure wave is emitted as a consequence of thermoelastic expansion. The amplitude of which is the stronger, the closer the pulse duration is to the stress confinement time of the absorber. The wave’s amplitude *P*(*t*) is proportional to the absorption coefficient, the pulse energy and the tissue’s Grüneisen parameter Γ(*T*), which strongly rises with temperature. The tissues Grüneisen parameter was approximated with a second order polynomial

(1) $$\Gamma \left(T\right)∼({\left(T^{2}-T_{0}^{2}\right)}^{}-2T_{m}\left(T-T_{0}\right))$$

using the zero-crossing *T*_0_ = −17.4 °C and the maximum *T_m_* = 102.9 °C as determined previously [16].

In order to extract an absolute temperature, the pressure amplitude must be calibrated to the tissue’s base temperature. This is done for each laser spot separately, since the absorbed laser energy varies locally owing to the tissue’s variations in absorption. Therefore, few probe pulses are applied just prior to tissue heating. In general, this method yields an averaged depth weighted and time dependent temperature *T_OA_(t)* over the irradiated volume with the measured pressure amplitude *P(t)* according to [18]

(2) $$T_{OA}\left(t\right)=T_{m}-\sqrt[{}]{{\left(T_{m}-T_{0}\right)}^{2}+\frac{P\left(t\right)}{S\cdot E_{p}}}$$

*E_p_* is the pulse energy and *S* the spot dependent calibration constant obtained for every irradiated site separately by calibrating the pressure amplitude to the body temperature just prior to heating. In order to obtain the temperature *T_max_(t)* at the RPE (*z* = 0) in the center of the beam (*r* = 0), which is the highest temperature in the tissue and therefore of particular interest, a time-dependent conversion function *f(t)* is needed, which is described in detail elsewhere [16].

(3) $$T_{max}\left(r=0,\;z=0,t\right)=f\left(t\right)\cdot T_{OA}\left(t\right)\;$$

The conversion function is approximated by a mono-exponential function

(4) $$f\left(t\right)=a_{1}+a_{2}\cdot e^{-\frac{t}{a_{3}}}\;$$

The coefficients were calculated for the RPE-choroidal model displayed below using a 200 µm beam diameter to *a*_1_ = 1.405, *a*_2_ = 3.186, *a*_3_ = 0.037. In order to support the measurements and to calculate the conversion function, temperature calculations by solving the heat diffusion equation were performed using the following model data and the thermal parameters of water:

- Spot diameter with top hat radiant exposure: 200 µm
- RPE’s absorption coefficient and thickness: µ_a_ = 575 cm^−1^, d = 6 µm
- Unpigmented layer: µ_a_ = 0, d = 4 µm
- Choroid’s absorption coefficient and thickness: µ_a_ = 4.2 cm^−1^, d = 400 µm

**Figure 2:**
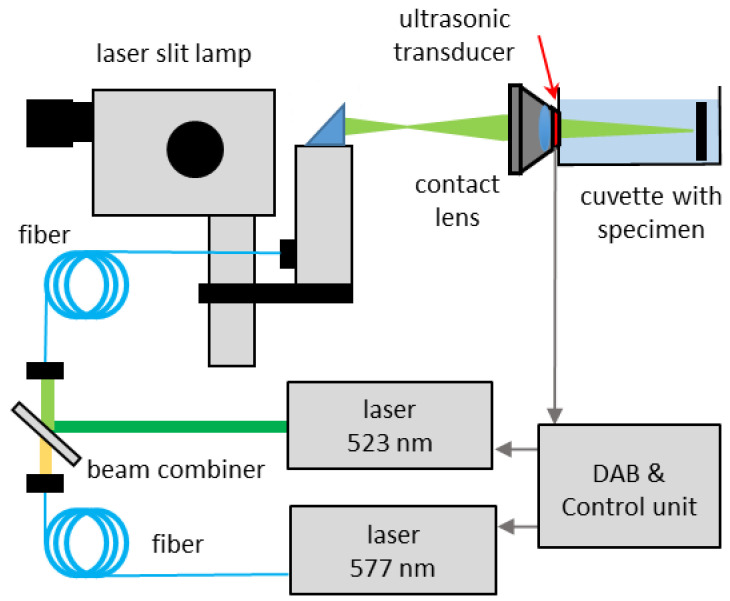
Experimental Set-up for optoacoustic temperature measurement. DAB: Data acquisition board.

The set-up for simultaneous temperature measurements is shown in Figure 2. Temperature probing was performed by a Q-switched frequency doubled Nd:YLF-laser at a wavelength of 523 nm (QG-523-1000, CrystaLaser Inc., Reno, NV, USA), emitting pulses with 75 ns full width at half maximum (FWHM) pulse duration. The repetition rate was adjusted to twice the repetition rate of the treatment laser system and the pulse energy was set to 5 µJ for all experiments at the laser slit lamp. The beams of the treatment laser and the probe laser were combined with a dichroic mirror and transmitted via standard 50 µm core diameter fiber (NA = 0.11) to the laser slit lamp (SL 130, Carl Zeiss Meditec AG, Jena, Germany). A contact lens (Mainster focal/grid lens, Ocular Instruments, Bellevue, WA, USA) with integrated ultrasonic transducer to record the pressure waves (modified at Medical Laser Center Lübeck, Lübeck, Germany), was mounted into a cuvette filled with saline solution and containing the specimen. The transducer voltage was amplified (Panametrics, Olympus, Tokyo, Japan), filtered with a bandpass (400 kHz–1.5 MHz) and recorded with a fast data acquisition board (CompuScope 8347, Gage Applied Technologies Inc., Lockport, NY, USA). For controlling the experiment, synchronizing the components and data processing an in house developed software written in LABVIEW (NI, Austin, TX, USA) was used.

## 3. Results

### 3.1. Laser Characteristics

**Figure 3:**
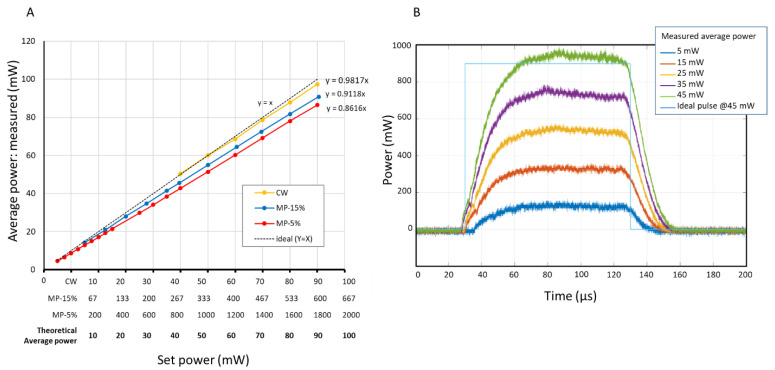
(**A**) Plots for the correlation between set laser power and the measured average laser power at the laser link for each mode. (**B**) Temporal profiles of a single laser pulse in micropulse mode with duty cycle of 5% for different average laser power settings.

The delivered power at the laser link showed slightly less than the set power, where the power drop was larger in MP modes (about 9% and 14% in MP-15% and MP-5%, respectively) than CW (2%) (Figure 3A). Typical single laser pulse shapes for different laser powers are shown in Figure 3B with a duration of 100 µs in the mode of MP-5% mode. The switch-on flank takes about 30 µs until a constant power is obtained. The switch off-flanks are slightly steeper than the on-flank and laser irradiation is completely off 20 µs after the switch-off time; thus, the full width at half maximum (FWHM) time is close to 100 µs as specified. The pulse repetition rate was measured to be 479.6 Hz instead of the nominal 500 Hz; thus, the exact DC was 4.8% instead of 5%.

### 3.2. Comparison of RPE Dead Cell Area (DCA)

**Figure 4:**
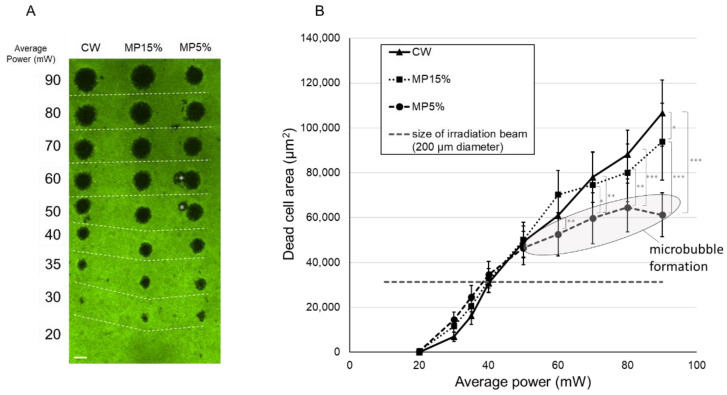
RPE cell viability assay after 200 ms irradiation with CW, MP with 15% DC and MP with 5% DC. (**A**) A representative image from calcein-AM viability assay 1 h after irradiation. Green fluorescence indicates live RPE cells, while non-fluorescence indicates dead or no RPE cells. Asterisk (*) in the image indicates an artifact caused by dead RPE cells at the irradiated site slightly displaced during the staining procedure. Scale bar = 200 µm (**B**) Dead area size for each setting, measured with the image analysis software FIJI. Mean values are plotted and standard deviations are displayed as error bars. Statistical analysis was conducted by one-way Analysis of Variance (ANOVA) with Tukey’s multiple comparison, using the statistic software GraphPad PRISM (Ver. 7.04). * *p* < 0.05, ** *p* < 0.01, *** *p* < 0.001.

Figure 4A shows the representative image of a calcein-AM viability staining 1 h after 200 ms irradiations. The non-fluorescent area indicates dead cell area (DCA). The calculation of ED50s determined that the average laser power at which RPE cells die with a 50% probability was calculated to be between 20 mW and 30 mW in all modes. Furthermore, the average laser power to destroy half the spot size area was also calculated to be 34.7 mW for CW, 32.4 mW for MP-15%, and 30.7 mW for MP-5%.

As shown in the graph of Figure 4B, there were statistically no differences in the damage areas with respect to average power among all modes up to 50 mW, where the DCA size rose almost linearly at a rate of 1600 μm^2^/mW. However, when it exceeds 50 mW, the slope at MP-5% dropped to about 610 μm^2^/mW (Figure 4B, ellipse). To note is that visible macrobubbles were occasionally observed through the slit lamp ophthalmoscope during irradiation in the MP-5% when the average power exceeds 50 mW (set output laser power ≈ 1000 mW), which suggests the concurrent thermomechanical RPE damage through microbubble formation resulting from exceeding the vaporization temperature at the melanosomes within a very short time range. Recorded OA signals also indicated the microbubble formation at some irradiations from average power of 50 mW in the MP-5% mode, as presented in detail later.

**Figure 5:**
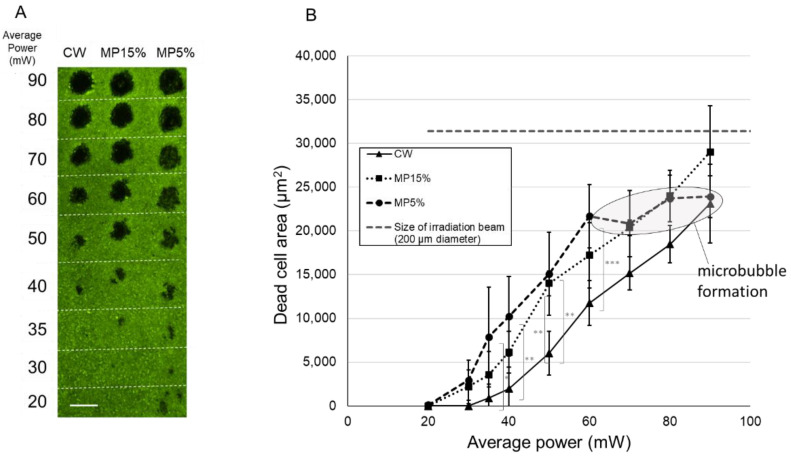
RPE cell viability assay after 20 ms irradiation with CW, MP-15% and MP-5%. (**A**) A representative image from calcein-AM viability assay 1 h after irradiation. Green fluorescence indicates live RPE cells, while non-fluorescence indicates dead or no RPE cells. Scale bar = 200 µm. (**B**) The size of dead cell area (DCA) for each mode and power setting, measured with the Image analysis software FIJI. Mean values are plotted and standard deviations are displayed as error bars. Statistical analysis was conducted by one-way Analysis of Variance (ANOVA) with Tukey’s multiple comparison, using the statistic software GraphPad PRISM (Ver. 7.04). * *p* < 0.05, ** *p* < 0.01, *** *p* < 0.001.

Figure 5A shows the representative image of calcein-AM viability staining 1 h after 20 ms irradiations. All DCAs were smaller than the irradiated spot size (31,400 µm^2^ area with 200 µm diameter, dashed line in Figure 5B) with the examined powers. The EC50 average laser power for the 50% probability of cell damage was calculated for 37 mW for CW, 27.5 mW for MP-15%, and 25 mW for MP-5%. Figure 5B shows that the DCA increased almost linearly with increased average power up to 90 mW with CW and MP-15%, whereas the slope was decreased with MP-5% at higher than 60 mW (Figure 5B, ellipse). At most average power settings up to 60 mW, DCA after MP-5% irradiations was significantly larger than after CW.

### 3.3. Optoacoustic Temperature Measurements

**Figure 6:**
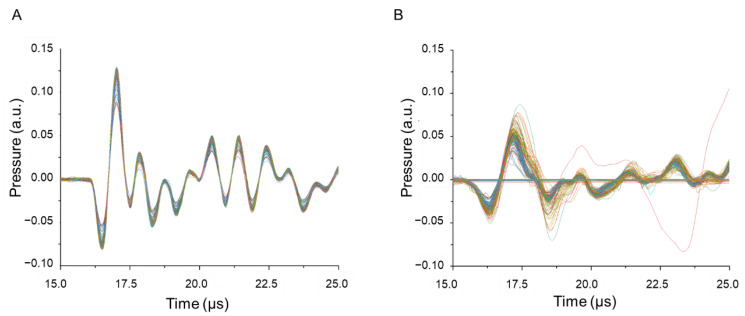
Representative pressure transients (OA signals) for absence (**A**) and presence (**B**) of microbubble formation, acquired during heating with 40 mW and 50 mW (MP-5% irradiation) respectively. Each transient is displayed in a different color for improved visualization of changes in amplitude and phase. (**A**) Absence of microbubble formation is indicated by phase stability with slight amplitude rise which accounts for slow and uniform heating. (**B**) Phase instability and rise in amplitude while pulse energy of probing laser remains constant, indicates microbubble formation.

The experiments for temperature measurements were carried out with the set-up shown in Figure 2. Due to the beam combining, additional fiber coupling and non-optimized optics of the laser slit-lamp used here instead of the laser link, a 40% loss of the laser powers for the 577 nm radiation was measured behind the slit-lamp compared to the setting at the micropulse console. Due to the measured pulse repetition rate of 479.6 Hz of the system, corresponding to a pulse-to-pulse period of 2.085 ms, the probe lasers repetition rate was set to the double frequency of 959.2 Hz in order to obtain two data points for each DC period. All experiments were carried out with a pulse energy of 5 µJ measured behind the slit-lamp. The probe laser was synchronized with the laser system when operated in the 5%-mode, such that its pulses were always applied at the end of the emission of a micropulse (at 99 µs after switch on of every micropulse) giving a peak pulse RPE temperature T_max,pulse_(t), and between two micropulses providing an intermediate pulse RPE temperature T_max,intermediate_(t). The probe laser was switched on 10 ms prior to the treatment laser for every irradiation spot in order to calibrate each locations absorption to the samples base temperature in the cuvette of 22 °C. Figure 6 shows examples of the measured acoustic transients of a single spot superimposed to each other for the MP-5% mode with average powers of (a) 40 mW and (b) 50 mW. Figure 6A shows a very reproducible and phase stable transients with monotonously increasing amplitude from which the temperature increase was calculated according to Equations (2) and (3). Figure 6B displays a reduced phase and amplitude stability and one strongly deviating transient. The amplitude and phase changes account for tissue changes and microbubble formation. Note the lower amplitudes and lower frequencies of the pressure transients in Figure 6B compared to Figure 6A even though the applied power was higher. This can likely be attributed to a laser spot site offside the acoustic axis of the transducer that leads to higher acoustic interference.

**Figure 7:**
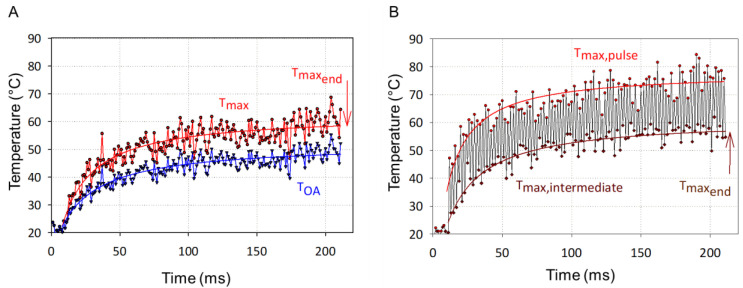
Representative temperature values for a single laser spot over an irradiation time of 200 ms measured in the CW mode and MP-5% mode with an average power of 25 mW on the same RPE tissue specimen, respectively, including the fitted average temperature rise for: (**A**) CW mode displaying the mean optoacoustic temperature (lower blue plot) and the maximal temperature (T_max_) course at the central RPE (upper red plot), (**B**) MP-5% mode showing the maximal temperature at the central RPE at the end of each micropulse (upper red plot: “T_max, pulse_”) and between two micropulses (lower brown plot: “T_max, intermediate_”).

Figure 7A,B show the representative measured temperature courses over the irradiation time of 200 ms in the CW mode and MP-5% mode, respectively, with an average power of 25 mW on the same RPE tissue.

The blue curve in Figure 7A shows the course of the mean optoacoustic volume temperature T_OA_(t) determined with the measured pressure amplitude P(t) by use of Equation (2) for the CW mode, while the red curve shows the peak temperature T_max_ on the beam axis at the RPE, calculated by means of Equation (3). Figure 7B shows the RPE temperature values T_max,intermediate_ measured at the end of each micropulse (red dots and red hyperbolic regression curve), and Tmax_end_ intermediately between two micropulses (brown diamonds and hyperbolic regression curve). The temperature showed a stepwise temperature increase, oscillating with a temperature difference of about 20 °C between the two measurement points.

**Figure 8:**
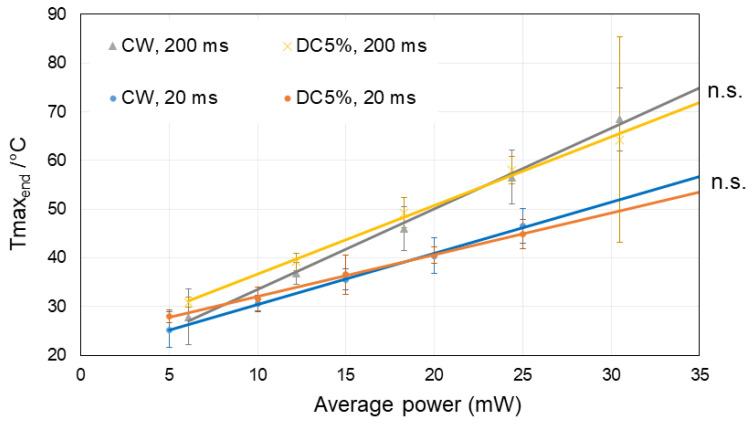
Comparison of the achieved maximal temperatures at the end of the irradiation time (Tmax_end_) between CW and MP-5% modes for 200 ms and 20 ms irradiations, respectively, for different average powers including the probe power up to 30 mW. There was no significant difference between CW and MP-5% for the same irradiation time. Mean values are plotted and standard deviations are displayed as error bars. Statistical analysis was conducted by one-way Analysis of Variance (ANOVA) with Tukey’s multiple comparison, using the statistic software GraphPad PRISM (Ver. 7.04). n.s.—not significant.

In Figure 8, the temperature at the end (Tmax_end_) of the irradiation periods of 20 ms and 200 ms, respectively, are shown for average laser powers of up to 30 mW for the CW and the MP-5% modes, including the 5 mW of the probe laser. The Tmax_end_ of both modes increase proportionally to the laser power as theoretically expected, no significant difference was found between the two modes. The temperature rises measured at the end of the irradiation are approximately 52% higher after 200 ms compared to 20 ms time.

**Figure 9:**
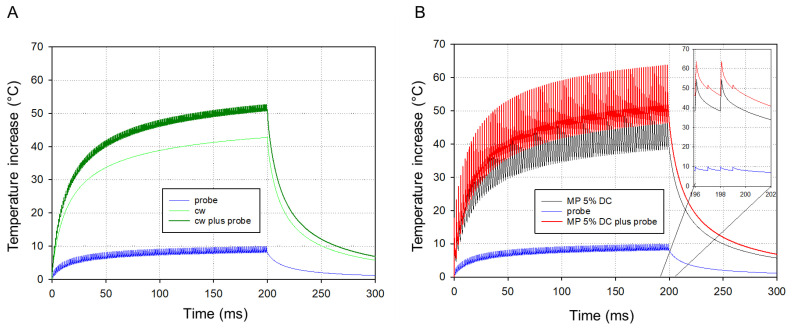
Simulations of the temperature increase during irradiation: (**A**) for the CW mode (25 mW) plus probe power (5 mW) and (**B**) for the MP mode with 5% DC at a peak power of about 500 mW, which leads to the same average laser power of 25 mW, plus probe power (5 mW). The inlay shows a magnification at the end of the irradiation.

The time evolution of the temperature increase was calculated for CW and MP modes, to be compared to experimental data. Figure 9A displays the theoretical temperature rise at the surface of the RPE in the center of the spot (z = 0, r = 0) when operating the system in CW mode with an average laser power of 25 mW, plus 5 mW from the probing laser (5 µJ per pulse applied with a repetition rate of 1 kHz). Since the temperature dependence of the tissue parameters were neglected, temperatures can be superimposed. With the assumed absorptions a temperature rises of 42 °C and 8.5 °C are achieved after a total irradiation period of 200 ms with the CW and probing laser irradiation, respectively, leading in total to a temperature rise of 50.5 °C (Figure 9A). As the temperature rise is proportional to the laser power applied, the data can be scaled proportionally to other powers. It should be noted that the temperature rise of a single probing laser pulse is just 0.8 °C in 75 ns and drops quickly.

Figure 9B displays the simulated temperature course for the MP-5% and a peak power of 500 mW, which leads to the same average laser power of 25 mW, plus 5 mW from the probing laser, as in Figure 9A, with a pulse repetition rate of 500 Hz. The micropulse-induced temperatures rise stepwise, with steps of about 12 °C. While the average rise is comparable to the CW case (cp. Figure 9A), the min and max values oscillate around the CW values, e.g., 47 °C and 63 °C are calculated after 198 ms (Figure 9B inlay), while the CW value is 50.5 °C.

## 4. Discussion

This study was conducted to compare the continuous wave mode with the micropulse mode (MP-5% and MP-15% DC) for different application times of 20 and 200 ms, respectively, when applying the same average laser power, and to analyze the data with respect to the achieved temperature rise and RPE damage area. In order to investigate the damage area, the calcein viability staining was used. For determining the temperature rise, optoacoustic temperature measurements were conducted during irradiation and the results were compared to temperature calculations.

### 4.1. Power Accuracy

For the comparative experiments among different modes, it is crucial to conduct them with the same actual average laser power for each mode. The laser system used in the current study showed a slight power reduction but stayed within the reference range of about 2% for CW, and 9% and 14% for MP-15% DC and MP-5% DC, respectively. One of the reasons for this slight power reduction in MP mode could be due the slower switch on-flank of the laser pulse compared to the switch off-flank. Note that such deviations may vary among different machines. To overcome this a more precise calibration between the set (average) power and the actual (average) power would be helpful. However, in clinical practice, the individual variations in eye transmission and retinal absorption are percentage wise much larger; thus, this inaccuracy would not play a major role.

### 4.2. Damage Mechanism

The duration of the single micropulse for the most MP laser treatment in clinical practice is chosen typically between 100 µs (5% DC) and 300 µs (15% DC). For pulse lengths in this range, the origin of damage at and close above threshold is thermal denaturation as shown in the literature [19,20,21]. However, the higher the peak power of a single pulse, the higher the temperature rise during pulse irradiation. If they reach the vaporization temperature, microbubbles nucleation at the strongly absorbing intracellular RPE melanin granule take place with the subsequent cellular disruption, as, e.g., shown by Neumann et al. [22]. Thus, thermal damage will be overlaid by thermomechanical disintegration at and around the RPE.

The smaller the duty cycle, the higher the power setting to obtain the same average power. It should be emphasized that the higher the power, the higher the instantaneous temperature at the melanosomes and the higher the probability of microbubble formation. In such cases, the risk of rupture of Bruch’s membrane or hemorrhage from choroidal vessels increases. Even though the power at which these events occur is much higher than the power used in clinical practice for the sublethal thermal therapy, it is necessary to make this fact known. Especially in non-minimally invasive laser irradiation, such as test irradiations for power titration and photocoagulation, low DC yields hardly any benefit but only increases the risk.

Last but not least, however, these pulse durations are much longer than the ones of a few microseconds to nanoseconds as used for selective RPE damage, such as in selective retina therapy (SRT, 1.7 µs) [23] or 2RT (2 ns) [24]. Even though microbubble formation is observed at high radiant exposure, the thermal background does not allow us to achieve selective RPE damage with the pulse durations of the MP-modes.

### 4.3. RPE Cell Damage: Threshold Power and Damage Extent

Wang et al. previously calculated the 3D temperature distribution and damage range with the Arrhenius model for continuous wave and micropulse laser irradiation and showed that tissue damage was mostly determined by the average power regardless of duty cycle. This has been verified in vivo experiments on rabbits [15]. In their reports, the average power threshold for the positive fluorescein angiography, indicating the damage of RPE tight junctions, was 22% and 35% lower for MP-5% than for CW at 200 ms and 20 ms irradiation time, respectively. However, the damage areas were very similar. The current findings in this study are consistence with those results. The onset of cell death was observed at a lower average power in the MP-5% than in the CW mode, and the difference was even larger at 20 ms. The reasons can be well explained by the slightly different temperature courses, the monotonous increase in the CW mode vs. the stepwise increase in the MP-modes with strong temperature peaks (Figure 7 and Figure 9). This leads to a slightly different temperature diffusion due to the nonlinearities of the heat diffusion equation and the Arrhenius damage integral, respectively, even though the same average power is applied.

Furthermore, microbubble formation was noticed when exceeding the threshold for damage by a factor of about 2 in the 5% DC MP-mode as observed in the experiments presented in Figure 4 and Figure 5, which is disclosed by the phase mismatch of the acoustic transients (Figure 6). In fact, optoacoustic pressure transients revealed microbubble formation starting from at average power of 50 mW for MP 5%. The onset of microbubble formation above the thermal damage threshold was also observed in our previous experiments performed with 2–50 µs pulses, which showed an increasing radiant exposure ratio of the onset of microbubble formation to cell damage threshold with increasing pulse duration [21]. The reason why the slope of the RPE cell damage (DCA) versus the laser power becomes smaller in this case can be explained by the facts that microbubbles reflect parts of the laser light before reaching the melanosomes shielded by the bubbles; thus, the overall absorption is reduced [22]. Further energy is needed for microbubble formation and growth with the associated pressure wave emission, which is consequently missed for thermal damage growth.

### 4.4. Temperature Measurements Methods

In addition to comparing the onset and extent of RPE cell death between both modes, this study also aims to measure and compare the transient temperature rise with an optoacoustic (OA) technique. Methods for measuring the temperature during laser irradiation using this technique have already been reported [17,25] and clinical data have been recorded during laser irradiation [18]. More recently, a temperature-controlled retinal laser therapy using feedback control irradiation is under development [16,26,27,28].

It was shown in the current study that the OA technique can also be applied in case of micropulse laser application. In order to enable temperature measurement with micropulse laser system, the probe laser irradiation was synchronized to the duty cycle. In order to verify the stepwise temperature increase as it has been calculated here and by Wang et al. [15], the frequency of the probe pulses was set twice as high as pulse repetition rate of the treatment laser. It gives a peak temperature T_peak_(t), and between two micropulses providing an intermediate temperature T_inter_(t). With the OA technique, it was experimentally proven for the first time that the MP application indeed resulted in the expected large temperature fluctuations and stepwise increase of the average temperature. There was almost no difference in average temperature rise among different modes for the same average power, as indicated previously [15] and in the simulation in the current study.

### 4.5. Temperature Results

The ED50 value of average power for RPE cell death at 200 ms irradiation were found to be between 20 mW and 30 mW for all modes, meaning that the Tmax_end_ was between 50–65 °C. Sramek et al. calculated the RPE damage threshold at 200 ms irradiation to be 53 °C using re-evaluated Arrhenius parameters [29]. Denton et al. found the threshold temperature of thermal RPE cell damage for cultured RPE cells to be 53 ± 2 °C at irradiation times from 100 ms to 1 s [30]. The temperatures measured in the current study cover the range determined by them using different technique in RPE cell cultures.

We can also compare the temperatures measured here on porcine organ explants with the ones measured in vivo on rabbits and clinically. For 20 ms CW irradiation, Schlott et al. reported ED50 temperature for ophthalmoscopic visibility at 61.7 °C [28]. The average laser power threshold for angiographic RPE cell damage at 20 ms CW laser irradiation is estimated to be about 85% of the threshold for ophthalmoscopic visibility (delayed visibility), according to the results of Wang et al. [15]. Taken together, the ED50 temperature of RPE cell death at 20 ms in the rabbit in vivo model is roughly calculated to be around 57 °C. In the current study, the ED50 average laser power for RPE cell death for 20 ms CW was about 37 mW, that is, about Tmax_end_ at 58 °C, which is consistent with the previous results-based estimation. On the other hand, for MP-5%, ED50 lies between 20 and 30 mW, that is, corresponding to the Tmax_end_ between 40 and 50 °C according to the current result. Referring again to the measurement by Wang et al., the ophthalmoscopic visibility depends on the average power regardless of the DC, however, angiographic visibility (RPE cell damage) showed a larger deviation from the ophthalmoscopic visibility at DC less than 15%, where the deviation is estimated about −40% for DC5% [15]. Based on this, the estimated threshold temperature for RPE cell death with MP-5% at 20 ms irradiation for rabbit in vivo model can be calculated to be around 51–54 °C. In the current study, the estimated threshold temperature for RPE cell death was even lower. According to these results, the threshold Tmax_end_ for RPE cell damage through MP-5% for 20 ms is likely to be below 54 °C, lower than that by CW.

Last but not least, first clinical data showed the visibility of mild coagulation following CW laser exposure on laser spot diameters of 100 and 300 µm, and irradiation times of 100 and 200 ms in the temperatures range between 70 °C and 85 °C. This also fits to the data due to the fact that a certain damage range is needed to become visible, and further regarding the reduced transparency and pathologic alterations of patient eyes [18].

### 4.6. Biological Effects CW and MP

What needs to be further elucidated is the difference in sublethal biological effects of different DCs, and compared to CW. Even though the average temperature increase is the same, the actual temperature lapse was shown to be different between CW and MP, in the measurement as well as in the simulation. Whether and which kind of biological difference can be caused in the irradiated RPE cells is still unclear. MP induces high temperature fluctuation in one irradiation, which might differently stimulate not only intracellular temperature sensors but also mechanical sensors at the cell plasma membrane, such as TRPV and PIEZO channels [31], causing a different calcium ion flux and biological responses during CW irradiation. Although this issue is currently unanswered yet to date, it is a crucial one for further clarification of the role of CW and MP in the treatment. CW at lower power can also be essentially applicable to achieve sublethal RPE hyperthermia. Sublethal RPE thermal irradiation is also possible with short duration CW irradiation as well, such as 10–20 ms, as in endpoint management [32] and irradiation with optoacoustic temperature control that is currently under development [33].

As described above, sublethal RPE hyperthermia has already been clinically proven to be effective in the treatment of several macular diseases such as CSC [5,6,7,8] and DME [9,10,11,12], and this applies to both MP [6,8,11] and CW [32]. Basic experiments have shown an increase in heat shock proteins [34] and subsequent various protective and anti-oxidative stress responses [35], the secretion of cytokines from RPE cells that may lead to improved retinal function [36] and the reduction of the thickness of Bruch’s membrane, suggesting inducing elimination of locally accumulated lipids [37]. These effects on RPE cells and Bruch’s membrane imply the further possible applicability of sublethal RPE hyperthermia to other diseases attributed to the dysfunction of RPE and Bruch’s membrane, such as early or intermediate age-related macular degeneration.

We believe that clearly demonstrating the similarities and differences between these modes, both in basic and clinical research, will lead to the development of a wider range of uses and, in turn, more patients will benefit from these minimally invasive treatments. Caution should be exercised in making such comparisons, however, by keeping the irradiation time and average power constant. To make comparisons under conditions in which they differ would not be a comparison of MP and CW, as the effects of differences in average temperature rise and irradiation time would be significant.

## 5. Conclusions

In conclusion, we showed the differences and similarities between CW and MP laser irradiations for RPE in terms of cell death incidence and temperature rise. At the same average power, there was no difference in the average temperature rise in RPE, and the degree of RPE cell death were almost the same. In addition, increasing the laser power at low DC was shown to increase a risk of mechanical damage. Measurements and simulations confirm that there is a difference in the course of temperature rise in both modes, with MP having higher temperature peaks and larger temperature fluctuation than CW. It is not yet clear how these differences may affect the biological response to the sublethal RPE thermal irradiation, which needs to be elucidated in further studies.
